# Supplementary material for: HSF-1 activates the ubiquitin proteasome system to promote non-apoptotic developmental cell death in C. elegans
Source: eLife. 2016 Mar 8;5:e12821. doi: 10.7554/eLife.12821 (PMC4821803; doi:10.7554/eLife.12821)
Supplement: Supplementary file 1. — (A) Wnt pathway genes and their effects on linker cell death. *All strains also contain the qIs56 reporter transgene to visualize the linker cell, except for the mom-2(ne834) and daf-21(p673) strains which contained the nsIs64 transgene. Allele numbers are in parentheses. † ± SEM. ‡Number of animals scored. ¶This strain also contained the unc-13(e1091) allele. §This strain had a severe migration defect which precluded accurate scoring of a survival phenotype. (B) RNAi against UPS components and effects on linker cell survival.*All animals contained rrf-3(pk1426); him-8(e1489) mutations and a qIs56 reporter transgene to visualize the linker cell. †LC, linker cell. ± SEM. ‡Number of animals scored. (C) RNAi against SCF components/BTB domain proteins and linker cell survival. *All animals contained rrf-3(pk1426); him-8(e1489) mutations and a qIs56 reporter transgene to visualize the linker cell. †LC, linker cell. ± SEM. ‡Number of animals scored. DOI: http://dx.doi.org/10.7554/eLife.12821.017 [file elife-12821-supp1.docx]

| **A. Wnt pathway genes and their effects on linker cell death** | | | |
| --- | --- | --- | --- |
| **Genotype^*^** | **Human homolog** | **% LC survival^†^** | **n^‡^** |
| *lin-44*(*n1792*) | Wnt | 2 ± 2 | 62 |
| *cwn-1*(*ok546*) | Wnt | 3 ± 2 | 91 |
| *cwn-2*(*ky756*) | Wnt | 0 | 67 |
| *mom-2*(*ne834*) | Wnt | 0 | 51 |
| *cwn-1*(*ok546*); *cwn-2*(*ky756*) | Wnt | 2 ± 2 | 80 |
| *egl-20*(*n585*) *cwn-2(ok895)* | Wnt | 54 ± 6 | 63 |
| *cwn-1*(*ok546*); *egl-20*(*n585*) | Wnt | MIG^§^ |  |
| *mig-1*(*e1787*) | Fz | 8 ± 2 | 179 |
| *cfz-2*(*ok1201*) | Fz | 2 ± 2 | 45 |
| *cam-1*(*gm122*) | Ror | 7 ± 3 | 73 |
| *lin-18*(*e620*) | Ryk | 6 ± 3 | 80 |
| *egl-20*(*n585*)*; lin-18*(*e620*) |  | 65 ± 5 | 112 |
| *mom-4*(*or39*)**^¶^** | MAPKKK7 | 0 | 31 |
| *mom-4*(*ne1539*) | MAPKKK7 | 3 ± 2 | 74 |
| *wrm-1*(*ne1982*) *lit-1*(*t512*) | β-catenin, NLK | 6 ± 3 | 66 |
| *sys-1*(RNAi) | β-catenin | 0 | 69 |
| *daf-16*(*mu86*) | FOXO | 1 ± 1 | 60 |
| *daf-21*(*p673*) | HSP90 | 0 | 48 |

| **B. RNAi against UPS components and effects on linker cell survival** | | |
| --- | --- | --- |
| **Genotype (RNAi)^*^** | **% LC survival**^†^ | **n^‡^** |
| **E2s** |  |  |
| *ubc-1* | 7 ± 3 | 100 |
| *ubc-2/let-70* | 45 ± 2 | 654 |
| *ubc-6* | 4 ± 2 | 118 |
| *ubc-7* | 3 ± 2 | 98 |
| *ubc-9* | 1 ± 1 | 86 |
| *ubc-12* | 7 ± 3 | 158 |
| *ubc-19* | 10 ± 3 | 96 |
| *ubc-20* | 2 ± 2 | 56 |
| *ubc-21* | 2 ± 1 | 102 |
| *ubc-22* | 2 ± 1 | 105 |
| *ubc-23* | 5 ± 2 | 115 |
| *ubc-24* | 3 ± 2 | 99 |
| *ubc-25* | 3 ± 2 | 89 |
| **HECT E3s** |  |  |
| *hecd-1* | 7 ± 2 | 115 |
| *D2085.4* | 6 ± 2 | 347 |
| *F36A2.13* | 2 ± 2 | 157 |
| *oxi-1* | 4 ± 2 | 95 |
| *wwp-1* | 2 ± 1 | 100 |
| *C02F5.7* | 6 ± 2 | 103 |
| **U-box E3s/E4s** |  |  |
| *cyp-4/mog-6* | 7 ± 3 | 100 |
| *ufd-2* | 2 ± 1 | 140 |
| *chn-1* | 3 ± 2 | 103 |
| *T10F2.4* | 3 ± 2 | 174 |
| **Monomeric RINGs** |  |  |
| *B0281.3* | 5 ± 2 | 80 |
| *B0281.8* | 0 ± 0 | 55 |
| *B0393.6* | 7 ± 3 | 94 |
| *B0416.4* | 3 ± 1 | 149 |
| *C01B7.6* | 4 ± 2 | 100 |
| *C01G6.4* | 8 ± 2 | 125 |
| *C06A5.8* | 3 ± 2 | 95 |
| *C06A5.9* | 3 ± 1 | 171 |
| *C11H1.3* | 4 ± 2 | 95 |
| *lin-41* | 2 ± 1 | 144 |
| *C16C10.5* | 2 ± 1 | 136 |
| *rnf-5* | 7 ± 2 | 105 |
| *C17E4.3* | 3 ± 2 | 105 |
| *C17H11.6* | 0 ± 0 | 96 |
| *C18B12.4* | 5 ± 3 | 39 |
| *C18H9.7* | 3 ± 2 | 101 |
| *C26B9.6* | 1 ± 1 | 84 |
| *C30F2.2* | 2 ± 1 | 105 |
| *C32D5.10* | 6 ± 2 | 105 |
| *C32D5.11* | 3 ± 2 | 105 |
| *C32E8.1* | 3 ± 2 | 80 |
| *C34E10.4*, *wrs-2* | 1 ± 1 | 94 |
| *C34F11.1* | 3 ± 2 | 105 |
| *C36A4.8*, *brc-1* | 9 ± 3 | 105 |
| *C45G7.4* | 4 ± 2 | 102 |
| *C49H3.5*, *ntl-4* | 0 ± 0 | 103 |
| *D2089.9* | 6 ± 4 | 35 |
| *F08B12.2*, *prx-12* | 5 ± 2 | 100 |
| *F08G12.5* | 4 ± 4 | 24 |
| *F10D7.5* | 1 ± 1 | 88 |
| *F10G7.10* | 1 ± 1 | 105 |
| *F11A10.3* | 2 ± 2 | 49 |
| *F16A11.1* | 0 ± 0 | 101 |
| *F35G12.9*, *apc-11* | 6 ± 3 | 85 |
| *F36F2.3* | 7 ± 2 | 120 |
| *F43G6.8* | 8 ± 3 | 101 |
| *F54B11.5* | 9 ± 3 | 98 |
| *nhl-1* | 5 ± 2 | 100 |
| *sli-1* | 6 ± 2 | 102 |
| *rfp-1* | 14 ± 3 | 100 |
| *T02C1.1* | 3 ± 2 | 104 |
| *sel-11* | 2 ± 1 | 105 |
| *apc-2* (APC E3) | 5 ± 3 | 63 |
| **Other** |  |  |
| *goa-1* (Ga protein) | 2 ± 1 | 100 |
| *asp-4* (aspartyl protease) | 1 ± 1 | 100 |
| *epn-1* (epsin homolog) | 5 ± 4 | 37 |
| *pbs-5* (proteasome subunit b5) | 2 ± 1 | 105 |
| *psa-4* (swi2/snf2 homolog) | 5 ± 2 | 102 |
| *bir-1* (IAP/BIR domain) | 5 ± 2 | 102 |
| *atg-7* (apg7p homolog) | 6 ± 2 | 100 |
| *otub-1* (deubiquitylase) | 6 ± 2 | 105 |
| *egl-27* (HDAC- NURD complex) | 2 ± 1 | 100 |
| *lin-40* (HDAC- NURD complex) | 5 ± 2 | 102 |
| *pdr-1(gk448)* (Parkin) | 5 ± 2 | 78 |

| **C. RNAi against SCF components/BTB domain proteins and linker cell survival.** | | |
| --- | --- | --- |
| **Genotype^*^** | **% LC survival^†^** | **n^‡^** |
| *cul-1*(RNAi) | 12 ± 1 | 304 |
| *cul-2*(RNAi) | 5 ± 1 | 240 |
| *cul-3*(RNAi) | 20 ± 4 | 257 |
| *cul-3*(LC-only RNAi) | 26 ± 1 | 194 |
| *cul-4*(RNAi) | 7 ± 2 | 289 |
| *cul-5*(RNAi) | 4 ± 4 | 243 |
| *cul-6*(RNAi) | 6 ± 5 | 262 |
| *skr-1* RNAi | 7 ± 4 | 41 |
| *skr-2* RNAi | 5 ± 2 | 100 |
| *skr-3* RNAi | 4 ± 2 | 105 |
| *skr-5* RNAi | 5 ± 2 | 110 |
| *skr-6* RNAi | 4 ± 2 | 114 |
| *skr-8* RNAi | 1 ± 1 | 77 |
| *skr-9* RNAi | 5 ± 2 | 81 |
| *skr-11* RNAi | 4 ± 2 | 78 |
| *skr-12* RNAi | 6 ± 2 | 103 |
| *skr-13* RNAi | 2 ± 2 | 73 |
| *skr-15* RNAi | 6 ± 3 | 87 |
| *skr-16* RNAi | 7 ± 3 | 99 |
| *skr-17* RNAi | 8 ± 3 | 89 |
| *skr-19* RNAi | 5 ± 2 | 95 |
| *skr-20* RNAi | 3 ± 2 | 70 |
| *skr-21* RNAi | 7 ± 2 | 108 |
| *kel-1* RNAi | 3 ± 3 | 106 |
| *kel-3* RNAi | 3 ± 2 | 75 |
| *kel-8* RNAi | 3 ± 2 | 97 |
| *eor-1* RNAi | 14 ± 2 | 197 |
| *bath-15* RNAi | 4 ± 2 | 75 |
| *bath-40* RNAi | 8 ± 3 | 74 |
| *mel-26* RNAi | 0 ± 0 | 71 |
| *bath-42* RNAi | 1 ± 1 | 73 |
| *ZC239.15* RNAi | 3 ± 2 | 66 |
| *btb-20* RNAi | 4 ± 2 | 72 |
| *D2045.8* RNAi | 7 ± 3 | 69 |
| *inso-1* RNAi | 5 ± 2 | 77 |
| *R12E2.1* RNAi | 7 ± 1 | 81 |
| *bath-43* RNAi | 4 ± 4 | 74 |
| *B2081.5* RNAi | 8 ± <1 | 75 |
| *kel-10* RNAi | 9 ± 3 | 74 |
| *F47D12.7* RNAi | 2 ± 2 | 85 |
| *tag-147* RNAi | 4 ± 2 | 71 |
| *spe-26* RNAi | 8 ± 3 | 77 |
| *C53A5.11* RNAi | 4 ± 2 | 76 |
| *C53A5.6* RNAi | Arrest at L3 | - |
| *C53A5.9* RNAi | 5 ± 2 | 87 |
