# Supplementary material for: HSF-1 activates the ubiquitin proteasome system to promote non-apoptotic developmental cell death in C. elegans
Source: eLife. 2016 Mar 8;5:e12821. doi: 10.7554/eLife.12821 (PMC4821803; doi:10.7554/eLife.12821)
Supplement: Supplementary file 2. — (A) Transgenic strain allele number and relevant plasmids. (B) Plasmid names, descriptions, and construction. DOI: http://dx.doi.org/10.7554/eLife.12821.018 [file elife-12821-supp2.docx]

**A. Transgenic Strains**

| Transgene | Constructs |
| --- | --- |
| *syIs187* | POPTOP::HIS-24-mCherry |
| *mhIs9* | *lin-17*::GFP |
| *wyEx806* | *lin-44*::GFP + *odr-1*p::GFP |
| *huIs7* | *hsp-16.2*p::ΔN-BAR-1*, dpy-20*(+)*, mec-7*::GFP |
| *muIs49* | *egl-20*::GFP*, unc-22*(+) |
| *sEx10067* | *hsp-1*p::GFP |
| *otIs198* | *hsp-16.2*p::NLS-mCherry |
| *nsIs230* | *hsp-16.41*p::mCherry |
| *deIs12* | 10.8kb-*bar-1*p::GFP |
| *muIs53* | *hsp-16.2*p::*egl-20 + unc-22*(*as*) |
| *nsEx4592* | pGO80[2.5kb *wrm-*1p::GFP], a gift of G. Oikonomou, 25 ng/μL + *unc-119*(+)*,* 75 ng/μL |
| *nsEx4584-5* | pMJK61[*mig-24*::*mig-5*B] (10 ng/μL) + pEB30[*lag-2*p::mCherry] (25 ng/μL) + *odr-1*p::RFP (25 ng/μL) + L4440, 40 ng/μL |
| *nsEx4589-90* | pMJK62[*mig-24*::*mig-1*B] (50 ng/μL) + pEB30[*lag-2*p::mCherry] (25 ng/μL) + *odr-1*p::RFP (25 ng/μL) |
| *nsEx4591* | pMJK62[*mig-24*::*mig-1*B] (25 ng/μL) + pEB30[*lag-2*p::mCherry] (25 ng/μL) + *odr-1*p::RFP (25 ng/μL) |
| *nsEx4586-8* | pMJK60[*mig-24*::HSF-1] (1 ng/μL) + pEB30[*lag-2*p::mCherry] (25 ng/μL) + *odr-1*p::RFP (25 ng/μL) + pBluescript, 49 ng/μL |
| *nsSi2-3* | MosSCI integrants of pOG124[*hsf-1*p::HSF-1(R145A)-GFP*]* |
| *nsIs346* | UV/TMP integration of *mhEx127*[5kb *mig-5*p::*mig-5*A-GFP + *unc-119*], a gift of M. Herman. |

| *nsIs495* | *lin-48*p::*mCherry* (25 ng/μL) + pBluescript (75 ng/μL), a gift of L. Kutscher. |
| --- | --- |
| *nsEx3669-72* | pJAZ042[*mig-24*p::*let-70* *C. briggsae* cDNA silently point mutated to avoid RNAi] + *unc-119(+)* |
| *nsEx3081* | *mig-24*p::*rde-1* cDNA::SL2::mCherry + *lag-2*p::mCherry |
| *nsEx2866,* | pJAZ002 (*let-70*(1.5kb)p::GFP) + *unc-119*(+) |
| *nsEx2864-5* | pJAZ001 (*let-70*(1.5kb)p::*let-70*::GFP + *unc-119*(+) |
| *nsEx3683-4* | pJAZ038[*mig-24*p::*let-70*(C35S) *C. briggsae* cDNA] + *unc-119*(+) |
| *nsEx4197,* | pJAZ117[*rbx-1*p::GFP] + *unc-119*(+) |
| *nsEx3950, 52-53, 94* | pJAZ068[*ubq-1*p::*ubq-1* first monomer::GFP] + *unc-119*(+) |
| *sEx10785* | *cul-3*p::GFP + *pCeh361* (*dpy-5* (+)) |
| *sEx10148* | *rpn-3*p::GFP + *pCeh361* (*dpy-5* (+)) |
| *nsEx5608* | *let-70-gfp* fosmid (WRM0627aA07) |
| *nsIs524-5* | pJAZ002-delHSE (*let-70*(ΔHSE)p::GFP) + *unc-119*(+) |
| *nsEx5153* | *mig-24*p::*btbd-2*(1ng/μL) + *lag-2*p::mCherry(30ng/μL) + pBluescript(70ng/μL) |
| *nsEx5154* | *mig-24*p::*btbd-2* (1ng/μL) + *lag-2*p::mCherry (30ng/μL) + *unc-119*(+) (70ng/μL) |

**B. Plasmids and plasmid construction**

| **Construct** | **Description** | **Notes** |
| --- | --- | --- |
| pMJK60 | *mig-24*p::*hsf-1* | A ~1.2kb *mig-24* promoter, a gift of E. Blum, was amplified as a *Pst*I/*Xma*I fragment using oligos 5’- ACACTGCAGTTTATCAGTTATCAGCAAGCAGAGAAATG -3’ and 5’- ACACCCGGGCGCCATTTTAATAAAATTGTGTAAGATG -3’ and subcloned into pTB4[*vap-1*::*hsf-1*::*unc-54* 3’UTR] |
| pMJK61 | *mig-24*p::*mig-5*B | *mig-5*B cDNA, a gift of H. Korswagen, was amplified as an *Xma*I/*Sal*I fragment using oligos 5’-AGTCCCGGGATGGAGCCGCCATGCAC-3’ and 5’-ATCGTCGACCTACTGTTCTCCCCGTCGAAATC-3’ and subcloned into pMJK60. |
| pMJK62 | *mig-24*p::*mig-1*B | *mig-24* promoter was amplified as an *Fse*I/*Asc*I fragment using oligos 5’- ACTGGGCCGGCCTTTATCAGTTATCAGCAAG-3’ and 5’- CCATGGCGCGCCTCATTTTAATAAAATTGTGTAAG-3’ and subcloned into pSM-SL2-mCherry, a gift of C. Bargmann. *mig-1*B cDNA, on pJRK135, also a gift from C. Bargmann, was amplified as an *Asc*I/*Eco*RI fragment using oligos 5’- TGAGGCGCGCCATGGGACCATTTCGTGGTTAC-3’ and 5’- ACTGAATTCTCAAATCATATTATTAGTTCGAAACGTC-3’ and subcloned into this plasmid. |
| pJAZ001 | *let-70*p::GFP in pPD95_75 | N2 genomic sequence was amplified using primers: 5’-GATTGGGCATGCTTTCCATATCTCTTG and 5’- GTTAGGGTACCCACATAGCGTACTTTTGCGTCC, and ligated into pPD95.75 (Andrew Fire) as an SphI/KpnI fragment. |
| pJAZ002 | *let-70*p::GFP in pPD95_75 | N2 genomic sequence was amplified using primers: 5’-GATTGGGCATGCTTTCCATATCTCTTG and 5’- CTTTTGGTACCCATGTTGGTGTTCTGATTTGCTG, and ligated into pPD95.75 (Andrew Fire) as an SphI/KpnI fragment. |
| pJAZ005 | *siah-1* RNAi in L4440 | cDNA library was amplified using primers: 5’- GCGCGGCCGCAAACGATGGCCAGAGTTCAG and 5’- GCGCGTCGACTCCAAGGTTTCCGTTTTCTG. This XbaI/XmaI fragment was ligated into L4440 (Julie Ahringer). |
| pJAZ006 | *let-70* RNAi in L4440 | The entire *let-70* cDNA sequence was amplified from a cDNA library using 5’- GCGCTCTAGATCCATTGGCAAGCTACGATT and 5’- GCGCGGTACCCCATTCTCTCGCCAATTGAT. This XbaI/KpnI fragment was ligated into L4440 (Julie Ahringer). |
| pJAZ008 | *let-70* 3’UTR RNAi in L4440 (RNAi C) | N2 genomic sequence was amplified using 5’- GGCGGCCGCGAGGCTAACACCATTCATACAAGA and 5’- GGGTCGACATGCAGTGGGCTCTTCAGTAATTTATTCAGGATTCTCAGACC. This NotI/SalI fragment was ligated into L4440. |
| pJAZ009 | *nhr-67/let-70* RNAi in L4440 | N2 genomic sequence was amplified using 5’- GGCCTCTAGAGGATCCTCTCCTAATTCGCC and 5’- GGCCCCCGGGTGTTGTGGTGGCTTGAACAT. This XbaI/XmaI fragment was ligated into an L4440 already containing *let-70* RNAi between sites HindIII/XhoI. |
| pJAZ012 | *set-16/let-70* RNAi in L4440 | N2 genomic sequence was amplified using 5’- GCGCTCTAGATGCAATCATAAAGCAGCGTCand 5’- GCGCCCCGGGGCGTCACTTGATCTGAG. This XbaI/XmaI fragment was ligated into an L4440 already containing *let-70* RNAi between HindIII and XhoI (*pJAZ021)*. |
| pJAZ013 | *nhr-67* RNAi in L4440 | N2 genomic sequence was amplified using 5’- GGCCTCTAGAGGATCCTCTCCTAATTCGCC and 5’- GGCCCCCGGGTGTTGTGGTGGCTTGAACAT. This XbaI/XmaI fragment was ligated into L4440. |
| pJAZ015 | *set-16* RNAi in L4440 | N2 genomic sequence was amplified using 5’- GCGCTCTAGATGCAATCATAAAGCAGCGTCand 5’- GCGCCCCGGGGCGTCACTTGATCTGAG. This XbaI/XmaI fragment was ligated into L4440. |
| pJAZ018 | *tir-1* RNAi in L4440 | N2 genomic sequence was amplified using 5’- GCGCTCTAGAATCGGATACGATGAAGCTGG and 5’- GCGCCCCGGGCCGATGAGTAATCAGCAGCA. This XbaI/XmaI fragment was ligated into L4440. |
| pJAZ019 | *tir-1/let-70* RNAi in L4440 | N2 genomic sequence was amplified using 5’- GCGCTCTAGAATCGGATACGATGAAGCTGG and 5’- GCGCCCCGGGCCGATGAGTAATCAGCAGCA. This XbaI/XmaI fragment was ligated into an L4440 already containing *let-70* RNAi between HindIII and XhoI (*pJAZ021)*. |
| pJAZ021 | *let-70* RNAi in L4440 | cDNA library was amplified using 5’- GCGCAAGCTTTCCATTGGCAAGCTACGATT and 5’- GCGCCTCGAGCCATTCTCTCGCCAATTGAT, and inserted into L4440 using HindIII/XhoI. |
| pJAZ024 | *rbx-1/let-70* RNAi in L4440 | N2 genomic sequence was amplified using 5’- GCGCTCTAGAATGGCCCAAGCAAGCGACAG and 5’- GCGCCCCGGGTTAGTGTCCGTACTTTTGGA. This XbaI/XmaI fragment was ligated into an L4440 already containing *let-70* RNAi between HindIII and XhoI (*pJAZ021)*. |
| pJAZ026 | *swd-2.2/let-70* RNAi in L4440 | N2 genomic sequence was amplified using 5’- GCGCTCTAGATTTGTTAATTCCGGGTTTGC and 5’- GCGCCCCGGGCGGAGCTTCCCACCATAATA. This XbaI/XmaI fragment was ligated into an L4440 already containing *let-70* RNAi between HindIII and XhoI (*pJAZ021)*. |
| pJAZ027 | *mCherry/let-70* RNAi in L4440 | N2 genomic sequence was amplified using 5’- GCGCTCTAGAAAGGGCGAGGAGGATAACAT and 5’- GCGCCCCGGGTTGACCTCAGCGTCGTAGTG. This XbaI/XmaI fragment was ligated into an L4440 already containing *let-70* RNAi between HindIII and XhoI (*pJAZ021)*. |
| pJAZ030 | *rpn-3* RNAi in L4440 | N2 genomic sequence was amplified using 5’- GCGCTCTAGAACAATGATCTTGCCCGCTAC and 5’- GCGCCCCGGGGGACAGGGATCTCGATTTGA. This XbaI/XmaI fragment was ligated into an L4440 vector. |
| pJAZ031 | *rpn-3/let-70* RNAi in L4440 | N2 genomic sequence was amplified using 5’- GCGCTCTAGAACAATGATCTTGCCCGCTAC and 5’- GCGCCCCGGGGGACAGGGATCTCGATTTGA. This XbaI/XmaI fragment was ligated into an L4440 already containing *let-70* RNAi between HindIII and XhoI (*pJAZ021)*. |
| pJAZ033 | *rbx-1* RNAi in L4440 | N2 genomic sequence was amplified using 5’- GCGCTCTAGAATGGCCCAAGCAAGCGACAG and 5’- GCGCCCCGGGTTAGTGTCCGTACTTTTGGA. This XbaI/XmaI fragment was ligated into an L4440 vector. |
| pJAZ035 | *lin-29* RNAi in L4440 | N2 genomic sequence was amplified using 5’- GCGCTCTAGAAGAGCACAGATTTTTGAACGAAG and 5’- GCGCCCCGGGATTTAATTTTACAGCCGATGGGT. This XbaI/XmaI fragment was ligated into an L4440 vector. |
| pJAZ036 | *lin-29/let-70* RNAi in L4440 | N2 genomic sequence was amplified using 5’- GCGCTCTAGAAGAGCACAGATTTTTGAACGAAG and 5’- GCGCCCCGGGATTTAATTTTACAGCCGATGGGT. This XbaI/XmaI fragment was ligated into an L4440 already containing *let-70* RNAi between HindIII and XhoI (*pJAZ021)*. |
| pJAZ038 | *mig-24*p::*let-70 briggsae* C85S | *pJAZ042* was mutagenized using Quikchange primers: 5’-ATTAACTCGAACGGATCGATTTCCCTCGATATTCTCCGATCACA and 5’- TGTGATCGGAGAATATCGAGGGAAATCGATCCGTTCGAGTTAAT using site-directed mutagenesis kit (Stratagene) |
| pJAZ040 | *mig-24*p::*let-70 elegans* cDNA | cDNA library was amplified using 5’- GCGCGGTACCGGTAGAAAAAATGGCTCTCAAAAGAATCCA and 5’- GCGCGAATTCCAGACGCTCTTCAGTAATTT. The KpnI/EcoRI fragment was ligated into a *mig-24*p::mCherry vector. |
| pJAZ042 | *mig-24*p::*let-70 briggsae* point mutated rescue | *let-70* cDNA carrying point mutations and flanked by KpnI/EcoRI was engineered via GeneArt Gene Synthesis (Life Technologies). The fragment was digested and inserted into a *mig-24*p::mCherry vector using KpnI/EcoRI. |
| pJAZ060 | *6xHis*::*let-70* in pET-28b(+) | pJAZ040 was used as a template for amplification using the primers 5’- GCGGGATCCATGGCTCTCAAAAGAATCCAG and 5’- GCGGCCGCTCACATAGCGTACTTTTGCG The fragment was ligated into *pET28b(+)* (Novagen) using BamHI and NotI. |
| pJAZ068 | *ubq-1*p::*ubq-*1::GFP | N2 genomic sequence was amplified using primers 5’-GCGCGCATGCCAGTTGCATGACGAGAAAAAAGA and 5’-GGTACCCCAATGTCTCCTCCGCGAAGACGAAGAACG. The SphI/KpnI fragment was cloned into pPD95.75. |
| pJAZ117 | *rbx-1*p::*GFP* | N2 DNA was amplified using primers 5’-CGCGGCATGCATGCTTATCATGCCTTTCTAGGTAAA and 5’-CGCGGGTACCGGTTTCTGGAAAAAATACAAATGAGAATAA. The SphI/KpnI fragment was inserted into pPD95.75 (Andrew Fire). |
| pJAZ151 | *cul-1*/*let-70* RNAi | *C. elegans* cDNA was amplified using primers 5’-CGCGTCTAGAGAAGACGGTCGAGCAGAATC and 5’- CGCGCCCGGGGCATGAATCCTGGCTCATTT. The XbaI/XmaI fragment was inserted into pJAZ021. |
| pJAZ119 | *let-70*(ΔHSE)::GFP | pJAZ002 was amplified using primers 5’- ATTTTATTAGTGTGACGTAACAGTTCACCACGAAGG and 5’- TTACGTCACACTAATAAAATAACTAACGATGAGAAATGATG. The fragment was DpnI digested and transformed. |
| pJAZ200 | *mig-24::btbd-2* cDNA | *C. elegans* cDNA was amplified using primers 5’-TAAAATGGCGGTACCATGAGCTCGGAAAATGGAG and 5’-CGCTCAGTTGGAATTCTCACTCGGCTGCTGTATAG. The fragment was Gibson cloned into *mig-24*p::mCherry digested with SacII/XmaI. |
